# Early and dynamic detection of doxorubicin induced cardiotoxicity by myocardial contrast echocardiography combined with two-dimensional speckle tracking echocardiography in rats

**DOI:** 10.3389/fcvm.2022.1063499

**Published:** 2023-01-13

**Authors:** Jun Zhang, Xin Li, Juan Liu, Yongning Shang, Lin Tan, Yanli Guo

**Affiliations:** Department of Ultrasound, Southwest Hospital, Army Medical University (Third Military Medical University), Chongqing, China

**Keywords:** doxorubicin, myocardial perfusion, global longitudinal strain, cardiotoxicity, myocardial contrast echocardiography

## Abstract

**Background:**

Anthracycline-induced cardiotoxicity is well-known as a side effect of chemotherapy. Currently, clinical imaging techniques are not capable to detect doxorubicin (DOX)-induced cardiotoxicity before a functional decline. The purpose of this study was to evaluate whether myocardial contrast echocardiography (MCE) can dynamically monitor the cardiac changes in the early stage in the DOX-induced rat model of cardiotoxicity.

**Methods:**

A weekly injection of 2.5 mg/kg of DOX was used to generate a rat model of cardiotoxicity. All groups underwent ultrasonic examinations including standard echocardiography, 2D speckle tracking echocardiography (2D-STE), and MCE. Then all rats were sacrificed immediately for histopathological evaluation.

**Results:**

A total of eight control rats and 32 DOX-treated rats were included in the study and grouped according to their treatment period. Decreased quantitative parameters of myocardial blood flow (MBF) (control vs. group 1: 133.31 ± 20.23 dB/s vs. 103.35 ± 21.60 dB/s, *P* = 0.048) and β (control vs. group 2: 11.17 ± 1.48/s vs. 7.15 ± 1.23/s, *P* < 0.001) were observed after 2 and 4 weeks of treatment, respectively, while left ventricular global strain (control vs. group 3: −23.67 ± 3.92% vs. −16.01 ± 3.40%, *P* = 0.002) decreased after 6 weeks of treatment and left ventricular ejection fraction (LVEF) (control vs. group 4: 82.41 ± 3.20% vs. 70.89 ± 9.30%, *P* = 0.008) decreased after 8 weeks of treatment. The main histopathological features are increased myocardial vacuolization and interstitial fibrosis and decreased myocardial microvessel density.

**Conclusion:**

Compared with standard echocardiography and 2D-STE, MCE can accurately and non-invasively detect changes in early myocardial perfusion, demonstrating the clinical potential of continuous and dynamic monitoring of DOX-induced cardiotoxicity.

## Introduction

Doxorubicin (DOX), a typical anthracycline, is generally used for treating numerous solid tumors, such as those of the breast, ovary and gastrointestinal tract, and hematological malignancies (1, 2). DOX treatment has greatly improved cancer survival rates; however, therapy with DOX can lead to cardiotoxicity, which affects the long-term outcomes of patients. Therefore, the early detection and prevention of DOX-induced cardiotoxicity are extremely important (3).

It is still unclear how DOX causes cardiac damage now. To date, many studies have been mostly focused on the effects of anthracyclines on myocardial function but little heed has been given to the effects on myocardial microcirculation perfusion. It is not clear whether microcirculation disorders occur before myocardial dysfunction. *In vitro* studies have shown that DOX exposure can lead to endothelial cell damage by increasing reactive oxygen species (ROS) production and directly mediating DNA damage (4–6). Additionally, Räsänen M et al. demonstrated that as DOX reduces the activity and release of key endothelial factors, it may promote the development and progression of cardiomyopathy (7). Interestingly, a recent study using a pig model showed that coronary microvascular toxicity occurred prior to any anatomical or functional impairment of the myocardium (8). Ideally, diagnostic tests should focus on myocardial microcirculation damage to diagnose early cardiotoxicity.

Left ventricular ejection fraction (LVEF), usually acquired by conventional echocardiography, is a routine and convenient indicator for monitoring cardiac toxicity during cancer treatment (9–11). In recent years, with the accumulating research on the myocardial strain, it has been confirmed that global longitudinal strain (GLS) is more sensitive than LVEF in early assessment of left ventricular (LV) systolic dysfunction and myocardial damage (12, 13). However, the diagnosis is usually made once LV functional impairment becomes apparent through either a decline in LVEF or GLS (11, 14). By this stage, it is often impossible to reverse myocardial damage. At present, prevention strategies cannot be developed due to the absence of validated early damage markers. Some studies have shown that the sensitivity and effect of cardioprotective therapy decrease gradually with the prolongation of the time interval between the occurrence of cardiotoxicity and the initiation of cardioprotective therapy (15). Therefore, it is important to develop effective strategies for detecting cardiac damage early and monitoring treatment-induced cardiotoxicity accurately.

By observing microbubble destruction and replenishment in the myocardium, myocardial contrast echocardiography (MCE) can non-invasively assess myocardial microcirculation perfusion (16). As coronary microcirculation blood flow and microvascular functional integrity are related to myocardial perfusion, MCE also evaluates the function of the complete coronary vascular system (17). Previous research has extensively found that MCE is not inferior to SPECT for the detection of myocardial perfusion (18, 19). Currently, MCE has been used to evaluate the myocardial activity and microcirculation perfusion function of coronary heart disease, diabetes, and hypertension in humans and animals (17, 20–22). To date, there has been a lack of MCE quantitative parameter studies through pretreatment, treatment, and overt LV systolic dysfunction, illustrating the full cycle of anthracycline treatment.

To further study the relationship between myocardial perfusion and the occurrence and development of DOX-induced cardiotoxicity, and to find imaging indicators for the early identification of cardiotoxicity, we applied MCE to the rat model of DOX-induced cardiotoxicity. We continuously and dynamically monitored the changes in relevant quantitative parameters of MCE before and during the treatment to observe obvious changes in LV systolic function and histopathology.

## Materials and methods

### Study design

**FIGURE 1:**
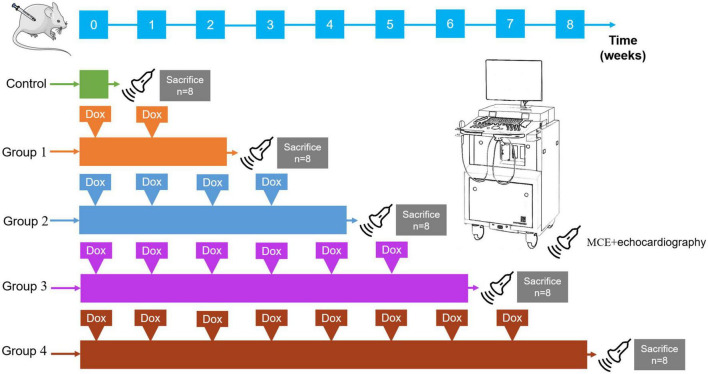
Study design. Rats received intraperitoneal DOX (2.5 mg/kg, once a week). Forty rats were randomly divided into the following five groups (8 rats in each group); control: 8 rats received normal saline; Group 1: 8 rats received two intraperitoneal injections of DOX; Group 2: 8 rats received four intraperitoneal injections of DOX; Group 3: 8 rats received six intraperitoneal injections of DOX; Group 4: 8 rats received eight intraperitoneal injections of DOX. MCE and echocardiography scans (probe) were performed in the long-axis planes in each group. All rats were sacrificed for a histopathologic evaluation immediately after the ultrasonic scan.

Animal care and use committee approval were obtained from Army Medical University for all experimental protocols. The study design is summarized in Figure 1. We obtained 40 adult male Sprague-Dawley (SD) rats (206.91 ± 9.30 g) from the medical experimental animal center of Army Medical University. During the experiments, food and water were freely available to all rats in an environment with a temperature control of 24°C and a light/dark cycle of 12 h. There were eight control rats that received weekly intraperitoneal injections of normal saline (2.5 mg/kg), the remaining 32 SD rats were randomly assigned into four DOX treatment groups (Groups 1–4): 2-, 4-, 6-, and 8-week treatment periods (cumulative dose: 5, 10, 15, and 20 mg/kg, respectively) with eight rats per group.

### Conventional echocardiography

During all echocardiographic examinations, volatile isoflurane (2.5% in oxygen, 500–700 ml/min) was used for anesthesia. The rats were first anesthetized in the induction box, and then placed in the supine position on the physiological information monitoring platform and an anesthesia concentration of 1–2% was maintained. Heart rate was kept constant at 350–370 beats/min and body temperature at 37°C throughout the experiment. Electrodes were fixed on the paws for continuous ECG monitoring and a shaver was used to remove anterior chest hair. The best parasternal long-axis view was selected, that is, the mitral valve, the aortic valve, and the maximum LV area were displayed.

This study used an ultrahigh resolution small animal imaging system (VEVO 2100; VisualSonics, Toronto, Canada) equipped with a linear array probe (MS400, VisualSonics) with a frequency of 24 MHz to acquire conventional transthoracic echocardiography images. When the papillary muscles are viewed in a parasternal long-axis view, LV wall thicknesses and chamber diameters and volume in end-diastole and end-systole, LVEF, LV fractional shortening (FS), and LV mass (LV mass) were determined by M-mode tracings. The flow velocity of the mitral valve was measured on the four chamber cardiac plane, the sampling frame was located on the level of the mitral valve, and the spectral Doppler image was obtained. The peak flow velocity (E), the isovolumic systolic time (ICT), the isovolumic diastolic time (IRT), and the LV ejection time (ET) were measured in the early diastole phase of mitral valve action, and tissue Doppler was measured at the junction of the ventricular septum and the mitral annulus to obtain the myocardial motion velocity (e’). The ratio of E to e’ (E/e’) and the Tei index, (ICT + IRT)/ET, were calculated (23).

### Two-dimensional speckle tracking echocardiography (2D-STE)

**FIGURE 2:**
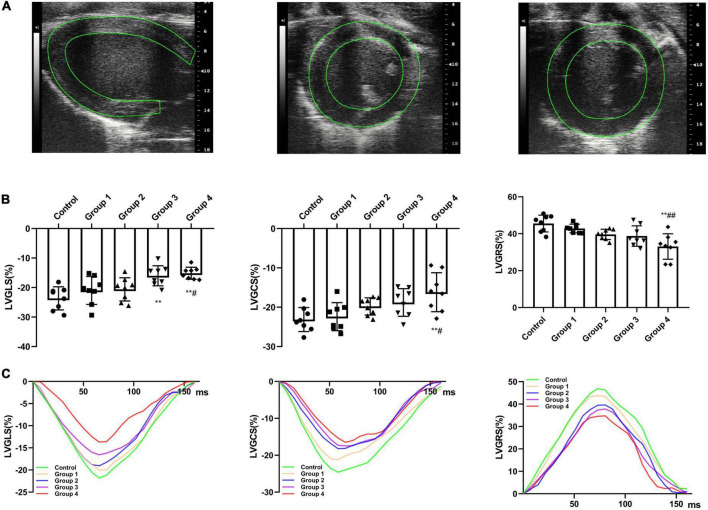
DOX treatment impaired cardiac strain. **(A)** Representative long-axis strain and short-axis strain images [Representative long-axis strain and short-axis strain images (for GCS and GRS analysis, respectively)]. **(B)** GLS, GRS, and GCS between control and DOX groups (*n* = 8). **(C)** Representative strain time curves of the five groups. LV, left ventricle; GLS, global peak longitudinal strain; GRS, global peak radial strain; GCS, global peak circumferential strain; The data are expressed as the means ± SDs. **p < 0.01 versus control, ^#^*p* < 0.05 versus Group 1, ^##^*p* < 0.01 versus Group 1.

By adjusting the scanning range, parasternal short- and long-axis views with the highest frame rate were recorded using B-mode to ensure the best possible visualization of the entire LV myocardium for 2D-STE analysis. Images of at least three cardiac cycles were acquired and analyzed using TomTec software (Philips Medical Systems, USA). The software automatically tracks the endocardial and epicardial boundaries and divides the equally spaced myocardial segments. The overall myocardial strain is the average value of each segment, excluding the images with poor tracking quality. In the parasternal long-axis plane, the LV global longitudinal strain (GLS) was obtained (Figure 2A); in the parasternal short-axis papillary muscles plane, the LV global circumferential strain (GCS) and global radial strain (GRS) were obtained (Figure 2A). Each value was averaged from three cine loops, and each loop was derived from three cardiac cycles.

### Myocardial contrast echocardiography (MCE)

The commercially available contrast agent Sonovue (Bracco, Italy), 59 mg/tube, was selected as the contrast agent. SonoVue consists of a suspension of microbubbles of stabilized SF6, which have an average size of 1–10 μm and a number of 2–5 × 10^8^/ml. Following the instructions provided by the manufacturer, the solution was prepared by diluting with 5 ml sodium chloride 0.9%. The contrast agent was continuously infused at a rate of 0.3–0.4 ml/min into a tail vein using a dedicated pump (Bracco, Italy) and a 24-gauge cannula (20).

For real-time MCE, we used a Philips EPIC 7 ultrasound diagnostic system (Philips Medical Systems, USA) and a linear array probe (eL18-4) with a frequency of 20 MHz. A set of pre- and post-processing settings was optimized for each rat: a focus was placed on the middle of the LV for the near field, the penetration depth was set to 2 cm, gains were adjusted to acquire images with no signal in the myocardium, and the dynamic range was set to maximum (60 dB). The above conditions were held constant for each experiment. In the myocardial contrast mode, the mechanical index (MI) was adjusted to 0.06, and the tail vein was continuously infused with SonoVue suspension. After filling the LV myocardium with a contrast agent, the “flash (high energy pulse, MI > 1.0)” was triggered to burst the ultrasound microbubbles in the myocardium, followed by automatic switching to the real-time low-energy contrast mode. The LV parasternal long-axis ultrasound cine loops were acquired, and continuous digital images of at least 20 cardiac cycles after flash were stored for quantitative analysis.

**FIGURE 3:**
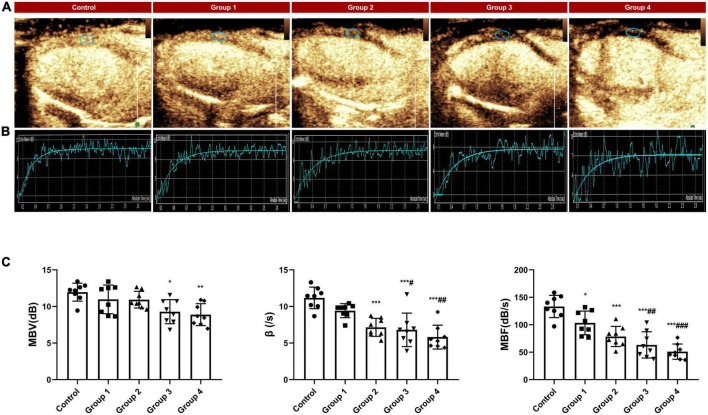
MCE assessed myocardial perfusion during DOX-induced cardiotoxicity development. **(A)** Representative MCE-based myocardial perfusion images of MCE in the five groups; **(B)** Representative time–intensity curves in the five groups, MBV = 11.31 dB, β = 12.56/s and MBF = 142.06 dB/s in control, MBV = 10.24 dB, β = 10.43/s and MBF = 106.80 dB/s in group 1, MBV = 10.63 dB, β = 7.86/s and MBF = 83.55 dB/s in group 2; MBV = 8.97 dB, β = 6.49/s and MBF = 58.21 dB/s in group 3; MBV = 7.24, β = 5.63/s and MBF = 40.76 dB/s in group 4; **(C)** The difference in MBV, wash in slope and MBF between the control and DOX groups. MBV, myocardial blood volume; MBF, myocardial blood flow. The data are expressed as the means ± SDs. *p < 0.05 versus control, **p < 0.01 versus control, ***p < 0.001 versus control; ^#^*p* < 0.05 versus Group 1, ^##^*p* < 0.01 versus Group 1, ^###^*p* < 0.001 versus Group 1.

The video was analyzed online or offline using QLAB (version 6.0, Philips healthcare) workstation. On the first frame image after “flash,” regions of interest were fixed on the middle segment of the LV anterior myocardium with an area of approximately 1.5 mm^2^ (Figure 3A) and manually tracked frame by frame to avoid the LV cavity and the right ventricular cavity. The first frame image after “flash” is set as the background frame and was fitted to an exponential function *Y* = *A* (1−*e*^−β^*^t^*) + *C* (24), in which A represents the peak intensity in the plateau phase or microvascular cross-sectional area, reflecting myocardial blood volume (MBV), β represents the rising slope of signal intensity, reflecting microvascular flow velocity, and the product of A and β reflecting myocardial blood flow (MBF) (25).

### Histopathology analysis

All rat was anesthetized and put into an enclosed CO_2_ gas chamber after undergoing MCE, then euthanized by thoracotomy and heart removal. The heart tissue was fixed in 4% neutral buffered formalin immediately. After being fixed for 48 h, the heart was continuously sectioned along the long axis of the LV papillary muscle (4 μm thick), which was similar to the MCE plane. Histomorphological changes in cardiac tissue were examined using hematoxylin and eosin (H&E) staining. The development of fibrosis in cardiac tissue was visualized using Masson’s trichrome staining. Myocardial vessels were assessed using CD31 staining (Abcam ab182981). The above pathological examination was evaluated by a pathologist who was blind to the ultrasonic results.

### Statistical analysis

The distribution of the data was assessed using the Shapiro–Wilk test, and the homogeneity of variance was evaluated using Levene’s test. The mean ± standard deviation is used to express continuous and normally distributed variables. The data were subjected to one-way ANOVA and Bonferroni *post hoc* test was performed for multigroup analyses. To assess the intra-observer and inter-observer variabilities in myocardial perfusion parameters, the intraclass correlation coefficient (ICC) and Bland-Altman statistics were used by means of two blinded examiners. Statistical significance was determined by a *p*-value of < 0.05. All data were analyzed using SPSS statistics version 23.0 (SPSS, Inc., Chicago, IL, USA) and plotted using GraphPad Prism software 8.0 (GraphPad Software, La Jolla, USA).

## Results

In this study, 40 rats were included in total. Eight rats were sacrificed immediately after ultrasound examination at baseline for pathological evaluation. The remaining rats were subjected to routine echocardiography, MCE, and pathological evaluation after 2, 4, 6, and 8 weeks of DOX intraperitoneal injection. After DOX injection, some rats exhibited arrhythmia, ascites, hepatomegaly, and diarrhea.

### Conventional echocardiography

The physiological and cardiac function measurements are presented in Table 1. As shown, the control group receiving saline injection stayed normal during the experiment, LV end-diastolic volume (LVEDV) was not changed while LV end-systolic volume (LVESV) was significantly increased at week 8 (85.66 ± 41.55 mm at baseline vs. 38.54 ± 8.32 mm in group 4, *P* = 0.11). FS was significantly decreased in 8-week DOX treatment groups (52.43 ± 3.51% at baseline vs. 42.03 ± 7.5% in group 4, *P* = 0.008), and LVEF was also reduced in group 4 (82.41 ± 3.20% at control vs. 70.89 ± 9.30% in group 4, *P* = 0.008). The DOX-treated rats showed a significantly higher LV mass (721.02 ± 82.58 mg at baseline vs. 930.56 ± 157.45 mg in group 4, *P* = 0.004) compared to control rats. Wall thickness had no significant difference between the control group and the DOX-treated groups. After 6 weeks of DOX treatment, E/e’ increased from 13.16 ± 1.46 at baseline to 16.23 ± 2.06 (*P* = 0.038). At 8 weeks, E/e’ increased to 16.46 ± 2.93 (*P* = 0.021). After DOX was administered for 6 weeks, the Tei index in the group of treated rats was significantly higher compared to the control group (0.49 ± 0.08 at baseline vs. 0.61 ± 0.06 in group 3, *P* = 0.019). After DOX was administered for 8 weeks, the Tei index was further increased to 0.66 ± 0.08 (*P* < 0.001).

**TABLE 1:** Physiological and conventional echocardiography data for all subjects.

	Control baseline (*n* = 8)	Group 1 2-week (*n* = 8)	Group 2 4-week (*n* = 8)	Group 3 6-week (*n* = 8)	Group 4 8-week (*n* = 8)
HR (/min)	368.25 ± 6.14	363.13 ± 3.72	364.25 ± 7.42	366.50 ± 11.51	361.50 ± 6.00
LVAWd (mm)	1.64 ± 0.06	1.72 ± 0.05	1.61 ± 0.15	1.71 ± 0.16	1.72 ± 0.18
LVPWd (mm)	1.70 ± 0.09	1.81 ± 0.10	1.76 ± 0.07	1.81 ± 0.17	1.81 ± 0.16
LVIDd (mm)	6.53 ± 0.34	6.58 ± 0.33	6.94 ± 0.61	7.01 ± 0.60	7.29 ± 0.60
LVIDs (mm)	3.11 ± 0.28	3.19 ± 0.28	3.76 ± 0.67	3.68 ± 0.76	4.26 ± 0.87**#
LVEDV (μL)	219.01 ± 25.55	222.99 ± 25.38	252.76 ± 50.99	258.10 ± 48.88	281.98 ± 50.79
LVESV (μL)	38.54 ± 8.32	41.14 ± 8.69	63.11 ± 27.32	60.76 ± 29.52	85.66 ± 41.55*#
EF (%)	82.41 ± 3.20	81.61 ± 3.01	75.75 ± 6.41	77.46 ± 7.17	70.89 ± 9.30**#
FS (%)	52.43 ± 3.51	51.55 ± 3.20	46.06 ± 5.95	47.86 ± 6.84	42.03 ± 7.55**#
LV mass (mg)	721.02 ± 82.58	788.30 ± 73.13	804.71 ± 85.86	866.36 ± 117.59	930.56 ± 157.45**
E/e’	13.16 ± 1.46	14.21 ± 1.68	15.16 ± 1.41	16.23 ± 2.06*	16.46 ± 2.93*
Tei index	0.49 ± 0.08	0.54 ± 0.08	0.58 ± 0.03	0.61 ± 0.06*	0.66 ± 0.08***##

HR, heart rate; LVAWd, left ventricular end-diastole anterior wall thickness; LVPWd, left ventricular end-diastole posterior wall thickness; LVIDd, left ventricular end-diastole diameter; LVIDs, left ventricular end-systole diameter; LVESV, left ventricular end-systolic volume; LVEDV, left ventricular end-diastolic volume; LVEF, left ventricular ejection fraction; FS, left ventricular fractional shortening; LV mass, left ventricular mass. *Control vs. Group subjects, *P* < 0.05; **Control vs. Group subjects, *P* < 0.01; ***Control vs. Group subjects, *P* < 0.001; ^#^Group 1 vs. Group subjects, *P* < 0.05; ^##^Group 1 vs. Group subjects, *P* < 0.01.

### 2D-STE

The LV strain parameters in the DOX treatment groups were significantly reduced than that of the control rats (Figure 2C). After DOX was received for 6 weeks, GLS decreased from −23.67 ± 3.92% to −16.01 ± 3.40% (*P* = 0.002) (Table 2 and Figure 2B). After DOX treatment for 8 weeks, GLS further decreased to −15.15 ± 2.05% (*P* = 0.001) (Table 2 and Figure 2B). Meanwhile, GCS and GRS also showed significant changes, GCS decreased from −23.14 ± 3.05% to −16.18 ± 4.94% (*P* = 0.004), and GRS decreased from 45.54 ± 4.55% to 33.09 ± 6.90% (*P* < 0.001) (Table 2 and Figure 2B).

**TABLE 2:** 2D-Speckle tracking data of all groups.

	Control (*n* = 8)	Group 1 (*n* = 8)	Group 2 (*n* = 8)	Group 3 (*n* = 8)	Group 4 (*n* = 8)
GLS (%)	-23.67 ± 3.92	-20.98 ± 4.73	-20.61 ± 3.94	-16.01 ± 3.40**	-15.15 ± 2.05**#
GCS (%)	-23.14 ± 3.05	-22.40 ± 3.57	-19.79 ± 2.19	-18.80 ± 3.52	-16.18 ± 4.94**#
GRS (%)	45.54 ± 4.55	42.82 ± 2.34	39.64 ± 2.77	38.76 ± 5.52	33.09 ± 6.90***##

GLS, global peak longitudinal strain; GRS, global peak radial strain; GCS, global peak circumferential strain. **Control vs. Group subjects, *P* < 0.01; ***Control vs. Group subjects, *P* < 0.001. ^#^Group 1 vs. Group subjects, *P* < 0.05; ^##^Group 1 vs. Group subjects, *P* < 0.01.

### MCE quantitative analysis

Compared with the control group, MBV did not begin to decrease significantly until 6 weeks after the DOX injection. However, the microvascular flow velocity and MBF in the DOX treatment group showed a gradual downward trend (Figure 3B). At 2 weeks, the myocardial perfusion parameter MBF significantly decreased (133.31 ± 20.23 dB/s in control vs. 103.35 ± 21.60 dB/s in group 1, *P* = 0.048) (Table 3 and Figure 3C). At 4 weeks, the microvascular flow velocity (11.17 ± 1.48/s in control vs. 7.15 ± 1.23/s in group 2, *P* < 0.001) (Table 3 and Figure 3C) and MBF (133.31 ± 20.23 dB/s at control vs. 78.64 ± 18.43 dB/s at group 2, *P* < 0.001) (Table 3 and Figure 3C) were decreased, and the difference was statistically significant. The difference in microvascular flow velocity and MBF between group 4 (8-week DOX-treated group) and group 1 (2-week DOX-treated group) were also significantly different (*P* < 0.05) (Table 3 and Figure 3C).

**TABLE 3:** Changes in perfusion parameters by MCE.

	Control (*n* = 8)	Group 1 (*n* = 8)	Group 2 (*n* = 8)	Group 3 (*n* = 8)	Group 4 (*n* = 8)
MBV (dB)	11.96 ± 1.23	10.97 ± 1.96	10.93 ± 1.14	9.29 ± 1.64*	8.88 ± 1.50**
β (/s)	11.17 ± 1.48	9.42 ± 0.96	7.15 ± 1.23***	6.81 ± 2.29***#	5.81 ± 1.63***##
MBF (dB/s)	133.31 ± 20.23	103.35 ± 21.60*	78.64 ± 18.43***	63.29 ± 23.93***##	51.04 ± 13.88***###

MBV, myocardial blood volume; MBF, myocardial blood flow. *p < 0.05 versus control, **p < 0.01 versus control, ***p < 0.001 versus control. ^#^*p* < 0.05 versus Group 1, ^##^*p* < 0.01 versus Group 1, ^###^*p* < 0.001 versus Group 1.

### Histologic results

**FIGURE 4:**
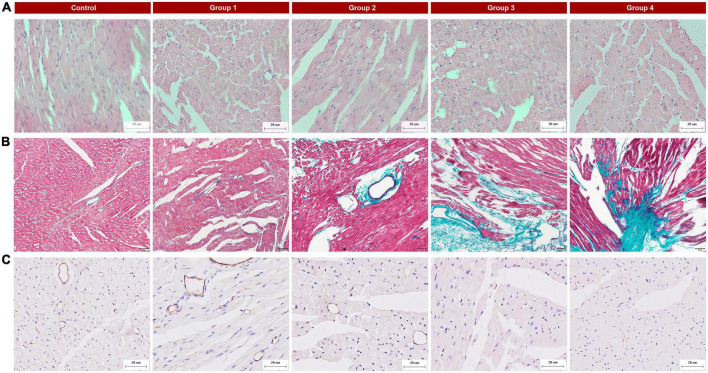
Typical histopathologic findings in the five groups. **(A)** Hematoxylin and eosin (HE) staining (×400): there were obvious vacuolar changes in the myocytes in the DOX treated group; **(B)** Masson’s trichrome staining (×200): myocardial interstitial fibrosis gradually increased and perivascular fibrosis worsened with the progression of DOX treatment; **(C)** CD31 staining (×400): reduced microvessel numbers in the DOX groups compared with the control.

Histopathological changes were found in the DOX-treated groups. HE staining of group 1 samples (collected at week 2, 5 mg/kg cumulative dose) displayed cardiomyocyte vacuolization (Figure 4A) without other obvious changes to myocardial tissue structure. With an increasing accumulated dose of DOX, the vacuolization and disorder of cardiomyocytes were more obvious, and the extracellular space was significantly increased (Figure 4A). In the control group, Masson staining showed that there were a small number of collagen fibers in the LV myocardial interstitium, but with prolonged DOX treatment time, the deposition of collagen fibers gradually increased (Figure 4B), especially perivascular fibrosis. In group 4, a large number of collagen fibers were deposited in the myocardial interstitium. CD31 immunohistochemistry suggested that the myocardial microvessel density gradually decreased with increasing accumulated doses of DOX (Figure 4C).

### Reproducibility of the quantitative analysis

**FIGURE 5:**
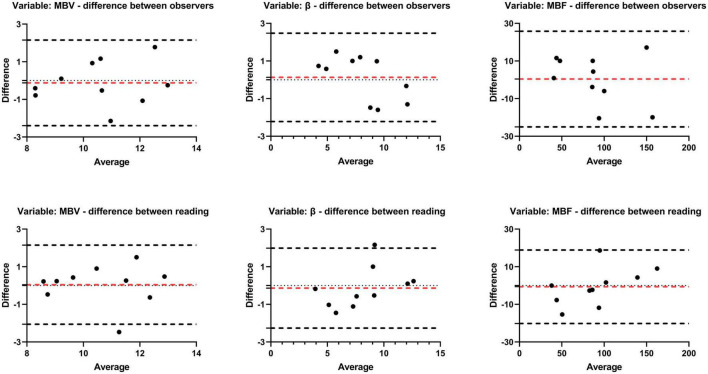
Bland-Altman agreement plots of perfusion parameters (MBV, β, MBF). Plots the difference between measurements of two reading or observers and mean measurements. The top and bottom lines show the 95% limits of agreement; the middle line (red) shows the mean difference.

A reproducibility analysis was conducted with 10 randomized rats. In terms of the intra-observer variability, there was an excellent ICC for MBV (0.808; 95% CI 0.428–0.948, *P* = 0.001), β (0.935; 95% CI 0.775–0.983, *P* < 0.001), and MBF (0.972; 95% CI 0.899–0.993, *P* < 0.001). Similar, although slightly worse, concordance values were found for the interobserver evaluation: MBV (0.796; 95% CI 0.399–0.944, *P* = 0.001), β (0.915; 95% CI 0.696–0.978, *P* < 0.001), and MBF (0.954; 95% CI 0.827–0.988, *P* < 0.001). The Bland-Altman analysis also presented a great agreement between intra-observer and inter-observer, as shown in Figure 5.

## Discussion

Anthracycline is prominent as an antitumor chemotherapy drug, but cumulative dose-related cardiotoxicity constrains its treatment applications in the clinic (26). The current clinical approach for detecting cardiotoxicity early is based on the abnormality of LV motion, known as LVEF or GLS (11, 14); Nevertheless, these changes are only evident at an early stage of the disease because they reflect severe damage to myocardial function. It has been proven that MCE as a promising quantitative method can detect subclinical myocardial changes in a variety of cardiomyopathies, but there is little research on the dynamic evaluation of myocardial perfusion during chemotherapy treatment using MCE. Our study combined 2D-STE and MCE to dynamically detect changes in LV structure, function, and myocardial perfusion during doxorubicin cycle treatment based on conventional echocardiography. This could be especially helpful to find new imaging markers for the early diagnosis and dynamic detection of DOX-induced cardiotoxicity in the clinic.

MCE was performed in a rat model of cardiotoxicity at different treatment periods, and the rats were immediately sacrificed to verify changes in histologic results. In this study, we found from using MCE that β and MBF were decreased at 4 and 2 weeks following treatment with DOX, respectively. However, GLS, E/e’, and Tei index were increased at 6 weeks, and EF and FS were reduced at 8 weeks. Accordingly, compared to either strain value or LVEF, β, and MBF had a higher diagnostic value and correlated well with the histologic findings.

In the current study, rats were treated for 2-, 4-, 6-, and 8- consecutive weeks with a dose of 2.5 mg/kg DOX once a week. Similar i.p. doses were also used in previous studies that have been shown to cause cardiotoxicity in rats (27, 28). Compared to the control group, an increase in E/e’ in the DOX group was observed at 6 weeks in our study, while the EF and FS of the DOX rats decreased at 8 weeks. Initially, diastolic dysfunction was observed, then systolic dysfunction. The significant alteration of these with DOX in this study is in accordance with a previous study (29). In addition, the Tei index is used to evaluate global cardiac function. Cardiac systolic dysfunction, ICT prolongation and ET shortening, diastolic dysfunction, and IRT prolongation and ET shortening can lead to an increase in the Tei index. The Tei index was increased at 6 weeks in our study, which may be related to cardiac diastolic dysfunction. In the DOX group, LVESV increased and LVEDV did not change significantly compared with the control group, suggesting a decrease in contractility, similar to that of cancer patients treated with DOX (30). However, an earlier study reported that LVESV was constant while LVEDV decreased in rats (31). In addition, LVEDV and LVESV were significantly increased after chemotherapy according to another animal study (32). There may be differences in results due to changes in sample sizes, body weight, dose factor, subject species, or treatment regimen.

In the echocardiographic assessment of LV function, myocardial strain, including GLS, GRS, and GCS can be used to quantify global and regional myocardial deformation or strain with less dependence on volume loading, size, scan angle, and geometry of the LV and with greater accuracy than the traditional measures of FS and EF (33). The American and European Societies of Echocardiography recently recommended routine 2D-STE as part of clinical follow-up for patients at risk for cardiotoxicity (34). In this study, 2D-STE was also used to evaluate DOX-induced cardiotoxicity. Rats experienced a significant decrease in GLS as early as the end of 6 weeks. In contrast, GRS, GCS, and LVEF kept stable and within normal ranges at that time point, which is similar to previous studies (35, 36). A number of reasons contribute to the longitudinal strain’s increased sensitivity in the detection of early cardiotoxicity compared with LVEF. In general, the subendocardial layer is always first affected by diseases. As the long-axis contraction of the myocardium depends primarily on fibers in this layer, a reduced longitudinal function is an early and accurate indicator of LV dysfunction, which is highly susceptible to ischemia, fibrosis, and hypertrophy (37–39). However, GLS did not respond to changes in cardiac function during the early stage, because relatively unaffected mid-myocardial and epicardial mechanics could compensate for abnormal GLS at least in the early stage, maintaining normal overall LV function.

MCE is a technique that can be used to evaluate myocardial tissue microcirculation perfusion in various ranges of situations. By using it, we can evaluate microcirculation perfusion in real time, estimate the volume of myocardial capillaries, and determine whether myocardial blood perfusion is well-distributed (40). MCE quantitative analysis suggested that microvascular flow velocity, MBF, and MBV were significantly decreased compared to controls. This indicated an increase in arteriolar resistance and a decrease in vasodilation of arterioles; myocardial microvessel density was decreased. Moreover, our results were similar to previous experiments in humans and rats. In cardiologic follow-up of breast cancer survivors, Gallucci G et al. (41) found that anthracycline drugs can lead to decreased myocardial perfusion without causing LV systolic dysfunction. SPECT showed that myocardial perfusion was significantly decreased in an anthracycline-induced cardiac toxicity model in rats (42). This may be attributable to DOX-induced oxidative stress, which can increase the production of ROS and nitric oxide (NO), leading to endothelial dysfunction and vasoconstriction of microvascular coronary arteries (43–46). Additionally, medial and adventitial layers of the microcirculatory coronary artery wall thickens, resulting in a general increase in microcirculatory arteriolar wall thickness (47). Vacuolization and swelling of cardiac myocytes, interstitial edema leading to the relative narrowing of the inner diameter of blood vessels, perivascular fibrosis, and decreased myocardial microvessel density may comprise one of the reasons.

In our model, differences in cardiac function, cardiac strain, and cardiac perfusion were detected between animals receiving different cumulative doses. Based on MCE, we observed that the change in myocardial perfusion precedes the change in LV strain against DOX-induced cardiotoxicity in a rat animal model. Our results extend observations to demonstrate an early imaging marker in DOX-induced cardiotoxicity, opening promising new prospects for the diagnosis and treatment of DOX-induced cardiotoxicity in patients treated with antitumoral chemotherapy.

In the present study, vacuolization of cardiomyocytes and increased myocardial interstitial fibrosis was observed in the DOX treated groups. Myocardial microvessel density was found to be significantly reduced according to histopathology. These results are consistent with those of previously published findings (8, 48–50).

## Limitations

There are some limitations in the present study. First, the DOX route of administration is not ideal because the present study used the intraperitoneal route rather than the intravenous route to treat patients with cancer. Second, unlike patients with cancer who develop DOX-induced cardiotoxicity, most of whom are elderly individuals, the rats used in this study are healthy and free of comorbidities. Finally, the pathophysiological process of DOX-induced early myocardial perfusion injury is still unclear. Furthermore, there is still much work to be done to determine the molecular and cellular mechanisms behind these findings.

## Conclusion

In the present study, we find that the earliest ultrasonic event according to MCE is the change in myocardial perfusion. Myocardial strain and cardiac function are normal at the early stage and do not change until later during the development of cardiotoxicity although deterioration of diastolic function, Tei index, and LVGLS occur prior to a substantial reduction in LVEF. Alterations in these parameters that reflect MCE may have the potential as a non-invasive technique in the evaluation of early and dynamic cardiac changes.

## Data availability statement

The raw data supporting the conclusions of this article will be made available by the authors, without undue reservation.

## Ethics statement

The animal study was reviewed and approved by the Institutional Animal Care and Use Committee at Army Medical University.

## Author contributions

JZ and XL contributed to the experiment, the data analyses, and wrote the manuscript. JL and LT contributed to the experiment. YS contributed to the study design and manuscript editing. YG contributed to the study design and manuscript writing—review and editing. All authors read and approved the final manuscript.
